# Supplementary material for: Latent classes associated with the intention to use a symptom checker for self-triage
Source: PLoS One. 2021 Nov 3;16(11):e0259547. doi: 10.1371/journal.pone.0259547 (PMC8565791; doi:10.1371/journal.pone.0259547)
Supplement: S1 Appendix — (DOCX) [file pone.0259547.s001.docx]

**S3 Appendix – Number of Participants Choosing Factors that are Important for Using a Symptom Checker for Self-Triage**

| **Factor** | **Number of participants** |
| --- | --- |
| 1. Your ability to perform **tasks on the computer** | 4 |
| 1. Your **self-rated health** | 6 |
| 1. Your **perceived accessibility** of symptom checkers | 8 |
| 1. Your **propensity or tendency** of using new technology | 2 |
| 1. Your **individual personality traits** | 3 |
| 1. Your perception of the **supports and resources** available to you | 2 |
| 1. Your **perception of risk** associated with using symptom checkers | 5 |
| 1. Your perspectives on the **perceived benefits** of using symptom checkers | 7 |
| 1. Your **trust** towards symptom checkers | 13 |
| 1. Your perspectives on the **effort expected** to use symptom checkers | 6 |
| 1. Your perception of the **credibility** of symptom checkers | 12 |
| 1. Your **social surroundings** | 2 |
| 1. Your perception of the **output quality** provided by symptom checkers | 7 |
| 1. Your perspectives on the **fun or pleasure** derived from using symptom checkers | 0 |
| 1. Your perception of the **tangibility of the result(s)** provided by symptom checkers | 8 |
| 1. Your perspectives on the **trade-off between costs and value** (applies if a fee is associated with the use of a symptom checker) | 6 |
| 1. Your perception on the symptom checker’s **compatibility** with your values, past experiences, and needs | 6 |
| 1. Your **habit** in adopting new technology | 3 |
| 1. Your level of **healthcare need** | 12 |

Based on a frequency analysis, the top five factors that seemed to be chosen most often are:

- **Trust** towards symptom checkers
- Perception of the **credibility** of symptom checkers
- Level of **healthcare need**
- Perception of the **tangibility of the result(s)** provided by symptom checkers
- **Perceived accessibility** of symptom checkers
