# Supplementary material for: Latent classes associated with the intention to use a symptom checker for self-triage
Source: PLoS One. 2021 Nov 3;16(11):e0259547. doi: 10.1371/journal.pone.0259547 (PMC8565791; doi:10.1371/journal.pone.0259547)
Supplement: S2 Appendix — (DOCX) [file pone.0259547.s002.docx]

**S1 Appendix – Survey**

**
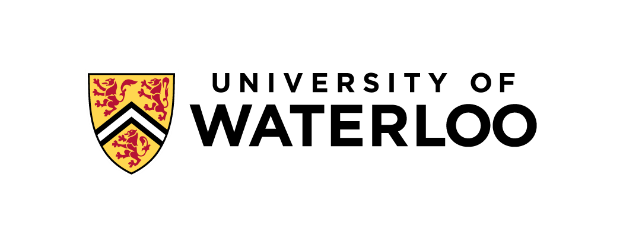
**

**Online Health Technology Survey**

**Introduction**

Thank you for participating in this survey. The purpose of this study is to understand university students’ perspectives on the use of artificially intelligent digital platforms for self-assessing their health and symptoms. The survey will take about 10 minutes to complete. In appreciation for the time given to this study, you can enter your name into a draw for a chance to win an iPadOS 14 valued at $429. Your odds of winning the prize is based on the number of individuals who participate in the study. We expect that approximately 500 individuals will take part in the study. The amount received is taxable. It is your responsibility to report this amount for income tax purposes.

All University of Waterloo students between the ages of 18 and 34 are eligible to participate. Demographic information such as gender and age will be collected to assess if differences in response exist based on this information. Information collected to draw for the prize will not be linked to the study data in any way, and this identifying information will be stored separately, then destroyed after the prize has been provided.

Participation in this survey is voluntary and you can decline to answer any question by leaving it blank. Withdrawing from the study does not disqualify you from the draw as you can, at any time, skip to the end of the survey to enter the draw. When provided to the researcher, the survey data will be anonymized so that no individual can be identified. The survey responses will be stored on a restricted access, secure server at the University of Waterloo and electronically archived for at least seven years. When information is transmitted over the internet, privacy cannot be guaranteed. There is always a risk your responses may be intercepted by a third party (e.g., government agencies, hackers). The SRC temporarily collects your computer IP address to avoid duplicate responses in the dataset.

There are no known or anticipated risks associated with participation in this study. Your participation in this study will help advance our knowledge on how digital platforms could potentially be used to reduce the burden on healthcare systems.

This study had been reviewed and received ethics clearance from the University of Waterloo Research Ethics Committee (ORE #41366). If you have questions for the Committee, contact the Office of Research Ethics, at 1-519-888-4567 ext. 36005 or ore-ceo@uwaterloo.ca. If you have questions about the study, please contact Stephanie Aboueid at [seabouei@uwaterloo.ca](mailto:seabouei@uwaterloo.ca).

By providing your consent, you are not waiving your legal rights or releasing the investigator(s) or involved institution(s) from their legal and professional responsibilities.

**I consent to participate in this survey**

1. Yes
2. No

**Section 1: Age and self-perceived health**

**SCREENER**

**Q1**

**How old are you?**

1. Younger than 18 years old **🡪 GO TO INELIGIBLE**
2. 18-24 years old
3. 25-29 years old
4. 30-34 years old
5. Older than 34 years old **🡪 GO TO INELIGIBLE**

**INELIGIBLE**

Thank you for your time but we are interested in interviewing University of Waterloo students aged 18-34.

New page

Artificially intelligent symptom checkers are smart digital platforms available online via desktop or through a mobile application. Users can enter a list of symptoms they are experiencing, and the app helps them identify whether or not they should seek medical care and provides a list of potential medical conditions that the user could be having. These platforms are typically free of charge and examples include **Babylon**, **Isabel**, and **mediktor** symptom checkers. These do not include the COVID-19 standard self-assessment tools.

[Placeholder of an image highlighting the typical process of a symptom checker]

**Q2a**

**In the past 12 months, have you used artificially intelligent symptom checkers to assess whether or not you needed to seek medical services?**

01 Yes – Code as Users for Section 4 questions

02 No – Code as Non-Users for Section 4 questions – **GO TO SECTION 2**

**New page**

**Q2b**

**In a typical year, how many times do you use artificially intelligent symptom checkers to assess whether or not you need to seek medical services?**

1. 0
2. 1-2
3. 3-5
4. More than 5

**Section 2**

**Preamble**

The first few questions will focus on your health and your use of healthcare.

**Q3**

**How would you describe your general health?**

1. Excellent
2. Very good
3. Good
4. Fair
5. Poor
6. Don’t know

**Q4**

**How often do you have someone help you read hospital materials?**

1. Always
2. Often
3. Sometimes
4. Occasionally
5. Never
6. I have never had to read hospital materials

**Q5**

**How often do you have problems learning about your medical condition because of difficulty understanding written information?**

1. Always
2. Often
3. Sometimes
4. Occasionally
5. Never
6. I don’t have a medical condition - **GO TO Q7**

**Q6**

**How often do you have a problem understanding what is told to you about your medical condition?**

1. Always
2. Often
3. Sometimes
4. Occasionally
5. Never

**Q7**

**How confident are you filling out medical forms by yourself?**

1. Always
2. Often
3. Sometimes
4. Occasionally
5. Never

**Q8a**

**In the 12 months before COVID 19 (March 1, 2020), did you see or talk to a family doctor or nurse about your physical, emotional or mental health? Please do not include any overnight stays in a hospital.**

1. Yes
2. No 🡪 **GO TO Q9**
3. Don’t know 🡪 **GO TO Q9**

**KEEP Q8b ON SAME PAGE**

**Q8b**

**How many times (in the past 12 months before COVID-19)?**

1. 0
2. 1-2
3. 3-5
4. More than 5

**Q9**

**When you need immediate care for a minor health problem, how long do you usually have to wait before you can have an appointment with a family physician or nurse?**

1. The same day
2. The next day
3. In 2 to 3 days
4. In 4 to 6 days
5. In 1 to 2 weeks
6. Between 2 weeks and one month
7. One month or more
8. Don’t know

**Q10**

**When you visit your primary care provider in their office, how often are you seen at your scheduled appointment time?**

- 1. Always 🡪 **GO TO Q12**
  2. Often
  3. Sometimes
  4. Rarely
  5. Never

**Q11**

**How long do you typically wait between the time of your appointment and the time you are seen by the primary care provider?**

1. Less than 15 minutes
2. 15 to less than 30 minutes
3. 30 minutes to less than one hour
4. 1 to less than 2 hours
5. 2 hours or more
6. Refuse to answer
7. Don’t know

**Q12**

**How many chronic health conditions do you have? We are interested in ‘long-term conditions’ which are expected to last or have already lasted 6 months or more and that have been diagnosed by a health professional. Some examples include asthma, diabetes, high blood pressure, heart disease.**

1. No chronic health conditions
2. 1 – 2
3. 3 – 5
4. 6 or more

**Section 3: Perspectives on artificial intelligence**

Artificial intelligence is defined as the theory and development of computer systems that can perform tasks that would normally require human intelligence. AI can be used to process a large set of data and its application in healthcare includes diagnosis and treatment recommendations, patient engagement, etc.

**Q13**

**I believe that the application of artificial intelligence in healthcare has a positive effect on healthcare services.**

1. Strongly disagree
2. Disagree
3. Neither disagree nor agree
4. Agree
5. Strongly agree
6. I don’t know

**Q14**

**I believe that the use of artificial intelligence leads to bias in healthcare because the data used may lead to predetermined ideas, prejudice or influence in a certain direction.**

1. Strongly disagree
2. Disagree
3. Neither disagree nor agree
4. Agree
5. Strongly agree
6. I don’t know

**Section 4: The use of symptom checkers**

**Preamble**

This section will assess your perspectives on the use of artificially intelligent health symptom checkers. These platforms ask for questions related to age, gender, symptoms, and some also ask about medical history. These platforms use artificial intelligence to help tailor the user experience by changing the question based on answers provided by users. The aim of these platforms is to help users identify whether or not they should consult a primary care provider based on the severity of their symptoms.

**FOR SECTION 4 ONLY:**

**IF Q2 = 02 (No) 🡪 DISPLAY ALL QUESTIONS IN BLUE AND BLACK**

- **THIS INCLUDES 🡪 Q15, Q16, Q18, Q19, Q21, Q23, Q25, Q27, Q28, Q30, Q32, Q34, Q36, Q38, Q40, Q42, Q44, Q46**

**IF Q2 = 01 (YES) 🡪 DISPLAY ALL QUESTIONS IN GREEN AND BLACK**

- **THIS INLCUDES 🡪 Q17, Q18, Q20, Q22, Q24, Q26, Q27, Q29, Q31, Q33, Q35, Q37, Q39, Q41, Q43, Q45, Q47**

**QUESTIONS IN BLACK (Q18, Q27) 🡪 DISPLAY FOR ALL**

**Q15**

**If available, I would try out an artificially intelligent symptom checker for self-triage (i.e., to determine if I need to see a healthcare provider or can manage my own health).**

1. Strongly disagree
2. Disagree
3. Neutral
4. Agree
5. Strongly agree

**Q16**

**If available, I would use an artificially intelligent symptom checker regularly for self-triage.**

1. Strongly disagree
2. Disagree
3. Neutral
4. Agree
5. Strongly agree

**Q17**

**I use an artificially intelligent symptom checker regularly for self-triage.**

1. Strongly disagree
2. Disagree
3. Neutral
4. Agree
5. Strongly agree

**Q18 🡪 DISPLAY FOR ALL**

**I would recommend an artificially intelligent symptom checker to a friend for self-triage.**

1. Strongly disagree
2. Disagree
3. Neutral
4. Agree
5. Strongly agree

**Q19**

**I would like to use an artificially intelligent symptom checker for self-triage.**

1. Strongly agree
2. Agree
3. Neutral
4. Disagree
5. Strongly disagree

**Q20**

**I like to use an artificially intelligent symptom checker for self-triage.**

1. Strongly agree
2. Agree
3. Neutral
4. Disagree
5. Strongly disagree

**Q21**

**An artificially intelligent symptom checker would be easy to use.**

1. Strongly agree
2. Agree
3. Neutral
4. Disagree
5. Strongly disagree

**Q22**

**An artificially intelligent symptom checker is easy to use.**

1. Strongly agree
2. Agree
3. Neutral
4. Disagree
5. Strongly disagree

**Q23**

**I would trust artificially intelligent symptom checkers with my health information.**

1. Strongly agree
2. Agree
3. Neutral
4. Disagree
5. Strongly disagree

**Q24**

**I trust artificially intelligent symptom checkers with my health information.**

1. Strongly agree
2. Agree
3. Neutral
4. Disagree
5. Strongly disagree

**Q25**

**I would be able to easily access artificially intelligent symptom checkers.**

1. Strongly agree
2. Agree
3. Neutral
4. Disagree
5. Strongly disagree

**Q26**

**I can easily access artificially intelligent symptom checkers.**

1. Strongly agree
2. Agree
3. Neutral
4. Disagree
5. Strongly disagree

**Q27 🡪 DISPLAY FOR ALL**

**The advantages of using artificially intelligent symptom checkers are obvious to me.**

1. Strongly agree
2. Agree
3. Neutral
4. Disagree
5. Strongly disagree

**Q28**

**Symptom checkers will provide me with high quality information.**

1. Strongly agree
2. Agree
3. Neutral
4. Disagree
5. Strongly disagree

**Q29**

**Symptom checkers provide me with high quality information.**

1. Strongly agree
2. Agree
3. Neutral
4. Disagree
5. Strongly disagree

**Q30**

**Artificially intelligent symptom checkers would perform well for self-triage.**

1. Strongly agree
2. Agree
3. Neutral
4. Disagree
5. Strongly disagree

**Q31**

**Artificially intelligent symptom checkers perform well for self-triage.**

1. Strongly agree
2. Agree
3. Neutral
4. Disagree
5. Strongly disagree

**Q32**

**Artificially intelligent symptom checkers would offer accurate information.**

1. Strongly agree
2. Agree
3. Neutral
4. Disagree
5. Strongly disagree

**Q33**

**Artificially intelligent symptom checkers offer accurate information.**

1. Strongly agree
2. Agree
3. Neutral
4. Disagree
5. Strongly disagree

**Q34**

**Artificially intelligent symptom checkers would offer up-to-date information.**

1. Strongly agree
2. Agree
3. Neutral
4. Disagree
5. Strongly disagree

**Q35**

**Artificially intelligent symptom checkers offer up-to-date information.**

1. Strongly agree
2. Agree
3. Neutral
4. Disagree
5. Strongly disagree

**Q36**

**Artificially intelligent symptom checkers would offer information relevant to my health context.**

1. Strongly agree
2. Agree
3. Neutral
4. Disagree
5. Strongly disagree

**Q37**

**Artificially intelligent symptom checkers offer information relevant to my health context.**

1. Strongly agree
2. Agree
3. Neutral
4. Disagree
5. Strongly disagree

**Q38**

**The information provided by the artificially intelligent symptom checker would reflect my health status.**

1. Strongly agree
2. Agree
3. Neutral
4. Disagree
5. Strongly disagree

**Q39**

**The information provided by the artificially intelligent symptom checker reflects my health status.**

1. Strongly agree
2. Agree
3. Neutral
4. Disagree
5. Strongly disagree

**Q40**

**I would be more likely to use an artificially intelligent symptom checker if I felt my personal health information was protected.**

1. Strongly agree
2. Agree
3. Neutral
4. Disagree
5. Strongly disagree

**Q41**

**I would be more likely to continue using an artificially intelligent symptom checker if I felt my personal health information was protected.**

1. Strongly agree
2. Agree
3. Neutral
4. Disagree
5. Strongly disagree

**Q42**

**Using an artificially intelligent symptom checker would help me assess the severity of my symptoms.**

1. Strongly agree
2. Agree
3. Neutral
4. Disagree
5. Strongly disagree

**Q43**

**Using an artificially intelligent symptom checker helps me assess the severity of my symptoms.**

1. Strongly agree
2. Agree
3. Neutral
4. Disagree
5. Strongly disagree

**Q44**

**Using an artificially intelligent symptom checker would be beneficial for my health.**

1. Strongly agree
2. Agree
3. Neutral
4. Disagree
5. Strongly disagree

**Q45**

**Using an artificially intelligent symptom checker is beneficial for my health.**

1. Strongly agree
2. Agree
3. Neutral
4. Disagree
5. Strongly disagree

**Q46**

**I would use an artificially intelligent symptom checker if it was endorsed by doctors or a health organization.**

1. Strongly agree
2. Agree
3. Neutral
4. Disagree
5. Strongly disagree

**Q47**

**I would continue using an artificially intelligent symptom checker if it was endorsed by doctors or a health organization.**

1. Strongly agree
2. Agree
3. Neutral
4. Disagree
5. Strongly disagree

**Section 5: Other questions**

The last few questions are about you and your household. The answers to these questions are used only for broad analysis purposes only. When analyzed, all of the data will be summarized, and the data will be anonymized so that no individual can be identified from these summarized results.

**Q48**

**What is your gender?**

1. Woman
2. Man
3. Non-binary
4. Prefer not to disclose
5. Prefer to self-describe ________________________

**Q49**

**What is your self-perceived racial or cultural group? (please select all that apply)**

1. First Nations, Inuit or Métis
2. White
3. South Asian (e.g., East Indian, Pakistani, Sri Lankan)
4. Chinese
5. Black
6. Filipino
7. Latin American
8. Arab
9. Southeast Asian (e.g., Vietnamese, Cambodian, Malaysian, Laotian)
10. West Asian (e.g., Iranian, Afghan)
11. Korean
12. Japanese
13. Other (please specify): ___________

**Q50**

**What is the highest level of education that you have completed?**

1. High school
2. Undergraduate degree
3. Master’s
4. PhD
5. Other, please specify: ______________

**Q51**

**What program are you currently enrolled in?**

1. Undergraduate degree
2. Master’s
3. PhD
4. Other (please specify): ____________

**Q52**

**In which faculty do you currently study at the University of Waterloo?**

1. Applied Health Sciences
2. Arts
3. Engineering
4. Environment
5. Mathematics
6. Science

**Q53**

**Are you currently employed?**

1. Yes
2. No 🡪 **GO TO Q55**
3. Prefer not to disclose 🡪 **GO TO Q55**

**Q54**

**How many hours (on average), do you currently work within a week period?**

1. 1 – 5 hours
2. 6 – 10 hours
3. 11 – 15 hours
4. 16 – 20 hours
5. 21 – 25 hours
6. 26 – 30 hours
7. 31 – 35 hours
8. 36 – 40 hours
9. 41+ hours

**Q55**

**How often do you have access to the Internet?**

1. Always
2. Often
3. Occasionally
4. Rarely
5. Never

**That is the end of the survey.**

**Q56**

**Please indicate below if you would like to be entered into a draw for a chance to win an iPad.**

- 1. Yes 🡪 **GO TO SEPARATE DRAW ENTRY PAGE**

1. No 🡪 **GO TO THANK YOU PAGE**

**Draw Entry Page:**

To be entered into the draw for a chance to win an iPad, please provide the information indicated below. Your name and contact information will be collected separately and will not be associated with your responses to the survey questions. Please see below for the Draw Rules and Regulations and the Privacy Policy.

First Name:

Last Name:

Phone Number:

Phone Extension:

E-mail address:

E-mail address (confirmation):

**Incentive Rules and Regulations**

**Incentive offered*:***For participating, you will be eligible for entry into a draw for a chance to win an iPad OS14 valued at $429. If you decide to withdraw your participation, you will still be eligible to enter the draw.

**Eligibility**: All university of Waterloo students between the ages of 18 and 34 enrolled in the Winter 2021 term who have completed the survey.

Eligibility to receive/win the prize begins on January 5, 2021, at 8:00 a.m. E.S.T. and closes on

February 15, 2021, at 11:59 p.m. E.S.T.

$429 iPad OS14 Draw: The winner of the iPad will be selected in a random draw from eligible entries on February 15, 2021. Your odds of winning the iPad will depend on the number of individuals who enter the draw. The award winner will be contacted by the researcher, Ms. Aboueid, by email (through the selected entrant’s UWaterloo e-mail address) within 10 business days of the draw. Before being awarded the iPad, the selected entrant must respond by the timeframe provided in Ms. Aboueid’s email (usually within 5 business days of being contacted by Ms. Aboueid) and correctly answer a time-limited skill-testing mathematical question. Failure to respond within the identified timeframe or a failed skill-testing question will result in the opportunity to win being passed on to the next eligible entrant.

**Please note:**

- The University is not responsible for any incomplete, failed or delayed transmission of your submission due to technical difficulties, including interruption or delays caused by equipment or software malfunction.
- If you have any questions about the survey, please contact Stephanie Aboueid at [seabouei@uwaterloo.ca](mailto:seabouei@uwaterloo.ca). If you have any technical problems with completing the online survey, please contact the Survey Research Centre by email at [srcccinb@uwaterloo.ca](mailto:srcccinb@uwaterloo.ca).

The University reserves the right to disqualify any entry not conforming to these Rules and Regulations at any time.  The University assumes no responsibility for entry fraud committed by an entrant and reserves the right to demand the return of the prize and all costs associated with remedying any prize awarded to an ineligible entry or entrant.

 Participation in the survey is confidential and voluntary. You may opt out of the survey at any time by closing the survey, with no loss of your chance to win the prize.

**Privacy Policy**

The Survey is being administered by the Survey Research Center on the behalf of a PhD candidate at the School of Public Health and Health Systems at the University of Waterloo. The purpose of the survey is to obtain information about university students’ perspectives related to the use of digital health technology for self-triage.

Only authorized staff tasked with analyzing and interpreting the results, and who have signed a data sharing and confidentiality agreement meant to ensure secure and appropriate handling of data, have access to the survey data.

Please note that when information is transmitted over the internet, privacy cannot be guaranteed.  There is always a risk your responses may be intercepted by a third party (e.g., government agencies, hackers). See [Waterloo’s guidelines on secure data transmission](https://uwaterloo.ca/information-systems-technology/about/policies-standards-and-guidelines/security/guidelines-secure-data-exchange-choosing-information) for more information.

***University of Waterloo Information, Privacy and Record Retention***

The University of Waterloo is committed to protecting your personal information and respecting the privacy of respondents.

All personal information is collected under the authority of the [University of Waterloo Act (1972)](https://uwaterloo.ca/secretariat/governance/university-waterloo-act) and will be processed in compliance with Ontario’s [Freedom of Information and Protection of Privacy Act](https://www.ontario.ca/laws/statute/90f31), as well as the University of Waterloo’s [Information and Privacy policies](https://uwaterloo.ca/privacy).

Questions about the collection, use, and disclosure of information associated with this survey should be directed to the Survey Research Center (519-888-4567 ext. 35071).  Questions about the collection, use, and disclosure of personal information by the university, should be directed to the Privacy Officer at [fippa@uwaterloo.ca.](mailto:fippa@uwaterloo.ca)

The collected data and reports will be managed according to the University records classification scheme, [WatClass](https://uwaterloo.ca/records-management/records-classification-and-retention-schedules), and will be securely destroyed when no longer needed by the University.

**Thank You Page**

Thank you for participating in the survey! Your feedback is extremely valuable. The draw winner will be selected and notified once the survey closes.

If you would like a copy of the results, please email the researcher at [seabouei@uwaterloo.ca](mailto:seabouei@uwaterloo.ca). They will be provided by 25/12/2021.

This study has been reviewed and received ethics clearance through a University of Waterloo Research Ethics Committee (ORE#41366). If you have questions for the Committee contact the Office of Research Ethics, at 1-519-888-4567 ext. 36005 or [ore-ceo@uwaterloo.ca](mailto:ore-ceo@uwaterloo.ca).

For all other questions or if you have general comments or questions related to this study, please contact Stephanie Aboueid, School of Public Health and Health Systems at [seabouei@uwaterloo.ca](mailto:seabouei@uwaterloo.ca) or Dr. Ashok Chaurasia, School of Public Health and Health Systems at [a4chaurasia@uwaterloo.ca](mailto:a4chaurasia@uwaterloo.ca).
