# Supplementary material for: Latent classes associated with the intention to use a symptom checker for self-triage
Source: PLoS One. 2021 Nov 3;16(11):e0259547. doi: 10.1371/journal.pone.0259547 (PMC8565791; doi:10.1371/journal.pone.0259547)
Supplement: S3 Appendix — (DOCX) [file pone.0259547.s003.docx]

**S2 Appendix – Construct Definitions and Source of Survey Questions**

| **Variable** | **Definition** | **Source** | **Corresponding Question(s) in the Survey (appendix 14)** |
| --- | --- | --- | --- |
| Perceived accessibility | *“Captures an individual’s perception of the ease or difficulty to gain access to or reach something.”* (Jung, 2008) | It has been argued that perceived accessibility is a powerful predictor of choice of information source rather than actual quality of the information (O’Reilly, 1982; Rice & Shook, 1988). | Question 25 |
| Trust | *“Willingness of a party to be vulnerable to the actions of another party based on the expectation that the other will perform a particular action important to the trustor, irrespective of the ability to monitor or control that other party”.* (Mayer et al., 1995, p. 712) | Gefen et al., 2003 demonstrated the importance of trust in understanding the acceptance of e-commerce.    Klein et al. (2007) demonstrated the importance of trust in the acceptance of health services online. | Question 23 |
| Perceived output quality | *“Judged by observing intermediate or end products of using the system, such as documents, graphs, calculations, and the like.”* (Davis et al., 1992) | Song et al. (2006) state that the quality of the information is the most important attribute for users seeking information.  Venkatesh & Davis (2000) outline that output quality positively influences perceived usefulness. In the case of e-health, this is the quality of the healthcare response given to the request; the health information obtained (Jung, 2008). | Questions 32, 34, 36, and 38 |
| Result demonstrability | *“Tangibility of the results of using the innovation.”* (Moore & Benbasat, 1991, p. 203; adapted by Venkatesh and Davis, 2000) | Venkatesh & Davis (2000) outline that result demonstrability is correlated with usage intentions. | Question 30 |
| Perceived ease of use (or effort expectancy) | *“degree to which a person believes that using a particular system is free of effort*” (David, 1989, p.320) | While this construct has been theoretically shown to influence technology use; a study conducted by Jung (2008) showed that perceived ease of use had limited effect on using e-health services – a reason for this however, may be that the services were not perceived difficult to use. | Question 21 |
| Perceived usefulness (or performance expectancy) | “*Degree to which a person believes that using a particular system would enhance his or her job performance*” (Davis 1989, p. 320) | Perceived usefulness has been shown to be a stronger determinant than perceived ease of use (Davis, 1989; Venkatesh and Davis, 2000; Jung, 2008) | Questions 18, 27, 28, 42, and 44 |
| Perspectives on AI in healthcare | Captures the perspectives of respondents on the use and application of AI in healthcare | This variable was added based on findings from the qualitative phase which suggests that use of AI-enabled symptom checkers may be influenced by perspectives on AI in general. | Questions 13 and 14 |
| Perceived credibility | “*The extent to which one believes that the other has the required expertise to perform effectively and reliably.”* (Lanseng & Andreassen, 2007, p.402). | This variable was identified to be an important factor in the qualitative phase of this work. | Questions 32, 34, and 46 |
| Self-perceived health | “…*a summary statement about the way in which numerous aspects of health, both subjective and objective, are combined within the perceptual framework of the individual respondent.”* (Tissue, 1972, p.93) | This construct may have an important influence on the use of self-service technology. For example, those who perceive their health to be poor may be more incline to use the technology (Kenny and Connolly, 2017). | Question 3 |
| Health literacy | “*Degree to which individuals have the capacity to obtain, process, and understand basic health-related decisions*.” (Institute of Medicine, 2004b) | Health literacy measures whether an individual is able to find health information, easily understand the information, use the information to make decisions, and make good choices about their lifestyle and healthcare (University of Arkansas for Medical Sciences, 2017). | Questions 4 to 7 |
| Healthcare use | Defined in this study as the frequency of using healthcare services (excluding overnight hospital stays) | Questions that measure whether healthcare services (excluding hospital stays) were used in the past 12 months and if so, the number of times healthcare services were sought (Statistics Canada, 2020). | Question 8A and 8B |
| Wait times | Captures wait times to schedule a medical appointment and at the medical appointment. | Questions that measure the number of days that the individual has to wait prior to getting an appointment with a family physician or nurse as well as the time that the individual waits at the office (Statistics Canada, 2020). | Questions 9 to 11 |
| Healthcare need | Defined as the need to use healthcare services due to an individual’s health condition(s). | A question that measures the number of “long-term health conditions” which are expected to last or already lasted 6 months (Statistics Canada, 2020). | Question 12 |
