# Supplementary material for: Latent classes associated with the intention to use a symptom checker for self-triage
Source: PLoS One. 2021 Nov 3;16(11):e0259547. doi: 10.1371/journal.pone.0259547 (PMC8565791; doi:10.1371/journal.pone.0259547)
Supplement: S4 Appendix — (DOCX) [file pone.0259547.s004.docx]

**S4 Appendix – Interpretation of the three- and four-latent class models**

For the model with three classes, the profile description of these classes is given in table 1. The first profile describes a group with positive attitudes towards various aspects of symptom checkers and were thusly labeled *tech acceptors.* The second group were the opposite, having a low probability of answering positively on any of the items assessed, and were labeled as *tech rejectors*. The third group had a mixed response pattern showcasing some negative perceptions, particularly related to trust, demonstrability, and output quality – this group was labeled as *skeptics*.

**Table 1. Three-latent-class model: Probability of positive perceptions for each subgroup**

|  | Latent Class (count; %) | | |
| --- | --- | --- | --- |
|  | Tech acceptors (756; 58%) | Tech rejectors  (110; 8%) | Skeptics  (439; 34%) |
| Trust | **0.5559** | 0.0644 | 0.1448 |
| Credibility | **0.9914** | 0.3127 | **0.8475** |
| Output quality | **0.8849** | 0.0789 | 0.4625 |
| Usefulness | **0.9456** | 0.0908 | **0.6356** |
| Demonstrability | **0.7362** | 0.1042 | 0.2484 |
| Accessibility | **0.7779** | 0.1806 | **0.7058** |
| Ease of use | **0.7179** | 0.2014 | **0.6375** |
| Perspectives about AI | **0.7466** | 0.3385 | **0.5292** |

Note: Item-response probabilities >0.5 are bolded to facilitate interpretation.

The four-class-model identifies similar groups as the three-class model (i.e., “*tech acceptors*”, “*tech rejectors*”, and “*skeptics*”) but includes an additional sub-group that had some positive perspectives related to the output but did not trust the platform and perceived it to be difficult to access and use – this group was labeled as “*unaware acceptors*”.

**Table 2. Four-latent-class model: Probability of positive perceptions for each subgroup**

|  | Latent Class (count; %) | | | |
| --- | --- | --- | --- | --- |
|  | Tech acceptors (578, 44%) | Tech rejectors  (138, 11%) | Skeptics  (360, 27%) | Unaware acceptors  (229, 18%) |
| Trust | **0.5480** | 0.0624 | 0.1300 | 0.4824 |
| Credibility | **0.9929** | 0.3765 | **0.8191** | **0.9813** |
| Output quality | **0.8835** | 0.0974 | 0.4149 | **0.8422** |
| Usefulness | **0.9726** | 0.1422 | **0.6073** | **0.8532** |
| Demonstrability | **0.7184** | 0.1046 | 0.2226 | **0.6743** |
| Accessibility | **0.9933** | 0.1499 | **0.8211** | 0.2892 |
| Ease of use | **0.8221** | 0.1935 | **0.7059** | 0.4598 |
| Perspectives about AI | **0.7626** | 0.3410 | **0.5340** | **0.6636** |

Note: Item-response probabilities >.5 are bolded to facilitate interpretation.
