# Supplementary material for: Latent classes associated with the intention to use a symptom checker for self-triage
Source: PLoS One. 2021 Nov 3;16(11):e0259547. doi: 10.1371/journal.pone.0259547 (PMC8565791; doi:10.1371/journal.pone.0259547)
Supplement: S5 Appendix — (DOCX) [file pone.0259547.s005.docx]

**S5 Appendix – Detailed GLM output**

**Table 1. Model with the five-class model as a predictor**

| **Model Fit Statistics** | | |
| --- | --- | --- |
| Criterion | Intercept Only | Intercept and covariates |
| AIC | 2594.716 | 2454.289 |
| SC | 2605.064 | 2506.029 |
| -2 Log L | 2590.716 | 2434.289 |

| **Type 3 Analysis of Effects** | | | |
| --- | --- | --- | --- |
| Effect | DF | Wald Chi-Square | Pr > ChiSq |
| Latent Class | 8 | 142.8164 | <.0001 |

| **Analysis of Maximum Likelihood Estimates** | | | | | | |
| --- | --- | --- | --- | --- | --- | --- |
| Parameter | Future Use | DF | Estimate | Standard Error | Wald Chi-Square | Pr > ChiSq |
| Intercept | Yes | 1 | -0.7758 | 0.2244 | 11.9535 | 0.0005 |
| Intercept | No | 1 | -0.3365 | 0.1952 | 2.9718 | 0.0847 |
| LC acceptors | Yes | 1 | 1.7149 | 0.2435 | 49.6191 | <.0001 |
| LC acceptors | No | 1 | -0.5237 | 0.2442 | 4.5978 | 0.0320 |
| LC skeptics | Yes | 1 | 1.0195 | 0.2848 | 12.8135 | 0.0003 |
| LC skeptics | No | 1 | 0.3365 | 0.2694 | 1.5600 | 0.2117 |
| LC tech seekers | Yes | 1 | 2.0163 | 0.2953 | 46.6259 | <.0001 |
| LC tech seekers | No | 1 | -0.4463 | 0.3594 | 1.5420 | 0.2143 |
| LC unsure acceptors | Yes | 1 | 0.7371 | 0.2760 | 7.1333 | 0.0076 |
| LC unsure acceptors | No | 1 | -0.6318 | 0.2900 | 4.7468 | 0.0294 |

| **Odds Ratio Estimates** | | | | |
| --- | --- | --- | --- | --- |
| Effect | Future Use | Point Estimate | 95% Wald Confidence Limits | |
| *Tech acceptors* vs. *tech rejectors* | Yes | 5.556 | 3.448 | 8.954 |
| *Tech acceptors* vs. *tech rejectors* | No | 0.592 | 0.367 | 0.956 |
| *Skeptics* vs. *tech rejectors* | Yes | 2.772 | 1.586 | 4.844 |
| *Skeptics* vs. *tech rejectors* | No | 1.400 | 0.826 | 2.374 |
| *Tech seekers* vs. *tech rejectors* | Yes | 7.510 | 4.210 | 13.397 |
| *Tech seekers* vs. *tech rejectors* | No | 0.640 | 0.316 | 1.294 |
| *Unsure acceptors* vs. *tech rejectors* | Yes | 2.090 | 1.217 | 3.590 |
| *Unsure acceptors* vs. *tech rejectors* | No | 0.532 | 0.301 | 0.939 |

**Table 2. Model with the five-class model as a predictor and confounders**

| **Model Fit Statistics** | | |
| --- | --- | --- |
| Criterion | Intercept Only | Intercept and covariates |
| AIC | 2594.716 | 2449.931 |
| SC | 2605.064 | 2574.106 |
| -2 Log L | 2590.716 | 2401.931 |

| **Type 3 Analysis of Effects** | | | |
| --- | --- | --- | --- |
| Effect | DF | Wald Chi-Square | Pr > ChiSq |
| Latent Class | 8 | 143.3710 | <.0001 |
| GenHealth | 2 | 2.7162 | 0.2572 |
| HL | 2 | 0.6488 | 0.7230 |
| HC Use | 2 | 5.6047 | 0.0607 |
| Wait time | 2 | 5.0084 | 0.0817 |
| Gender | 4 | 5.8547 | 0.2103 |
| Race | 2 | 12.3150 | 0.0021 |

| **Analysis of Maximum Likelihood Estimates** | | | | | | |
| --- | --- | --- | --- | --- | --- | --- |
| Parameter | Future Use | DF | Estimate | Standard Error | Wald Chi-Square | Pr > ChiSq |
| Intercept | Yes | 1 | -0.6534 | 0.5069 | 1.6617 | 0.1974 |
| Intercept | No | 1 | -1.0934 | 0.6537 | 2.7979 | 0.0944 |
| LC acceptors | Yes | 1 | 1.7233 | 0.2462 | 48.9998 | <.0001 |
| LC acceptors | No | 1 | -0.5710 | 0.2498 | 5.2239 | 0.0223 |
| LC skeptics | Yes | 1 | 0.9614 | 0.2866 | 11.2564 | 0.0008 |
| LC skeptics | No | 1 | 0.3250 | 0.2747 | 1.3999 | 0.2367 |
| LC tech seekers | Yes | 1 | 2.0371 | 0.2980 | 46.7329 | <.0001 |
| LC tech seekers | No | 1 | -0.4118 | 0.3640 | 1.2799 | 0.2579 |
| LC unsure acceptors | Yes | 1 | 0.7322 | 0.2777 | 6.9519 | 0.0084 |
| LC unsure acceptors | No | 1 | -0.6206 | 0.2947 | 4.4364 | 0.0352 |
| GenHealth Good | Yes | 1 | 0.0190 | 0.2034 | 0.0088 | 0.9255 |
| GenHealth Good | No | 1 | 0.4466 | 0.2867 | 2.4273 | 0.1192 |
| HL High | Yes | 1 | -0.0398 | 0.1966 | 0.0409 | 0.8396 |
| HL High | No | 1 | 0.1759 | 0.2818 | 0.3895 | 0.5326 |
| HC Use | Yes | 1 | 0.3023 | 0.1342 | 5.0731 | 0.0243 |
| HC Use | No | 1 | 0.0593 | 0.1773 | 0.1118 | 0.7381 |
| Wait time Short | Yes | 1 | -0.2901 | 0.1585 | 3.3492 | 0.0672 |
| Wait time Short | No | 1 | -0.4003 | 0.1993 | 4.0337 | 0.0446 |
| Gender Men | Yes | 1 | -0.0570 | 0.3919 | 0.0211 | 0.8844 |
| Gender Men | No | 1 | 0.4993 | 0.5242 | 0.9074 | 0.3408 |
| Gender Women | Yes | 1 | -0.00540 | 0.3875 | 0.0002 | 0.9889 |
| Gender Women | No | 1 | 0.1584 | 0.5206 | 0.0926 | 0.7610 |
| Race White | Yes | 1 | -0.0184 | 0.1509 | 0.0149 | 0.9028 |
| Race White | No | 1 | 0.5676 | 0.1871 | 9.2065 | 0.0024 |

| **Odds Ratio Estimates** | | | | |
| --- | --- | --- | --- | --- |
| Effect | Future Use | Point Estimate | 95% Wald Confidence Limits | |
| *Tech acceptors* vs. *tech rejectors* | Yes | 5.603 | 3.458 | 9.078 |
| *Tech acceptors* vs. *tech rejectors* | No | 0.565 | 0.346 | 0.922 |
| *Skeptics* vs. *tech rejectors* | Yes | 2.615 | 1.491 | 4.586 |
| *Skeptics* vs. *tech rejectors* | No | 1.384 | 0.808 | 2.371 |
| *Tech seekers* vs. *tech rejectors* | Yes | 7.669 | 4.276 | 13.752 |
| *Tech seekers* vs. *tech rejectors* | No | 0.662 | 0.325 | 1.352 |
| *Unsure acceptors* vs. *tech rejectors* | Yes | 2.080 | 1.207 | 3.584 |
| *Unsure acceptors* vs. *tech rejectors* | No | 0.538 | 0.302 | 0.958 |
| GenHealth good vs. Poor or do not know | Yes | 1.019 | 0.684 | 1.518 |
| GenHealth good vs. Poor or do not know | No | 1.563 | 0.891 | 2.741 |
| HL high vs. low or average | Yes | 0.961 | 0.654 | 1.413 |
| HL high vs. low or average | No | 1.192 | 0.686 | 2.071 |
| HC Use yes vs. no or do not know | Yes | 1.353 | 1.040 | 1.760 |
| HC Use yes vs. no or do not know | No | 1.061 | 0.750 | 1.502 |
| Wait time short vs. medium or long | Yes | 0.748 | 0.548 | 1.021 |
| Wait time short vs. medium or long | No | 0.670 | 0.453 | 0.990 |
| Gender men vs. other | Yes | 0.945 | 0.438 | 2.036 |
| Gender men vs. other | No | 1.648 | 0.590 | 4.603 |
| Gender women vs. other | Yes | 0.995 | 0.465 | 2.126 |
| Gender women vs. other | No | 1.172 | 0.422 | 3.250 |
| Race white vs. non-white | Yes | 0.982 | 0.730 | 1.320 |
